# Inhibition of Rho-associated coiled-coil containing protein kinase enhances the activation of epidermal growth factor receptor in pancreatic cancer cells

**DOI:** 10.1186/1476-4598-10-79

**Published:** 2011-07-03

**Authors:** Masanori Nakashima, Seiji Adachi, Ichiro Yasuda, Takahiro Yamauchi, Junji Kawaguchi, Toshimasa Hanamatsu, Takashi Yoshioka, Yukio Okano, Yoshinobu Hirose, Osamu Kozawa, Hisataka Moriwaki

**Affiliations:** 1 Department of Gastroenterology, Gifu University Graduate School of Medicine, Gifu 501-1194, Japan; 2 Department of Pharmacology, Gifu University Graduate School of Medicine, Gifu 501-1194, Japan; 3 Department of Molecular Pathobiochemistry, Gifu University Graduate School of Medicine, Gifu 501-1194, Japan; 4 Division of Pathology, Gifu University Hospital, Gifu 501-1194, Japan

**Keywords:** ROCK, EGFR, cell proliferation, pancreatic cancer

## Abstract

**Background:**

Rho-associated coiled-coil containing protein kinase (Rho-kinase/ROCK) is involved in various cellular functions including cell proliferation, and is generally considered to be oncogenic, while some studies show that ROCK functions as a negative regulator of cancer progression. As a result, the precise role of ROCK remains controversial. We have previously reported that Rho-kinase/ROCK negatively regulates epidermal growth factor (EGF)-induced cell proliferation in SW480 colon cancer cells. In the present study, we investigated the role of ROCK in EGF receptor (EGFR) signaling in the pancreatic cancer cell lines, Panc1, KP3 and AsPc1.

**Results:**

In these cells, Y27632, a specific ROCK inhibitor, enhanced EGF-induced BrdU incorporation. The blockade of EGF stimulation utilizing anti-EGFR-neutralizing antibodies suppressed Panc1 cell proliferation. EGF induced RhoA activity, as well as the phosphorylation of cofilin and myosin light chain (MLC), both targets of ROCK signaling, and Y27632 suppressed both of these processes, indicating that the phosphorylation of cofilin and MLC by EGF occurs through ROCK in Panc1 cells. EGF-induced phosphorylation of EGFR at tyrosine residues was augmented when the cells were pretreated with Y27632 or were subjected to gene silencing using ROCK-siRNA. We also obtained similar results using transforming growth factor-α. In addition, EGF-induced phosphorylation of p44/p42 mitogen-activated protein kinase and Akt were also enhanced by Y27632 or ROCK-siRNA. Moreover, an immunofluorescence microscope study revealed that pretreatment with Y27632 delayed EGF-induced internalization of EGFR. Taken together, these data indicate that ROCK functions to switch off EGFR signaling by promoting the internalization of the EGFR.

**Conclusions:**

While EGF first stimulates the activation of the EGFR and subsequently increases cancer cell proliferation, EGF concurrently induces the activation of ROCK, which then turns off the activated EGFR pathway via a negative feedback system.

## Introduction

Pancreatic cancer is a common malignancy, ranking thirteenth in incidence, and eighth as the cause of cancer-related death worldwide [1]. Surgical resection is the only curable treatment at present, but only 10-15% of patients are able to undergo surgery at the time of diagnosis. Most pancreatic cancer has already reached an advanced stage when the first symptoms appear. Furthermore, it is difficult to diagnose pancreatic cancer at an early stage, even with advanced medical imaging techniques such as computed tomography and magnetic resonance imaging.

The standard treatment for patients with advanced pancreatic cancer is chemotherapy. Gemcitabine has been the standard of treatment during the last decade, but the median survival of patients treated with gemcitabine is only 5-6 months. Many clinical trials have failed to demonstrate any improvement in overall survival with the addition of different drugs to gemcitabine [2]. Therefore, the development of new treatments for unresectable pancreatic cancer is required.

The epidermal growth factor receptor (EGFR) is a member of the ErbB family of receptor tyrosine kinases [3]. Binding of ligands such as epidermal growth factor (EGF) [4] or transforming growth factor-α (TGF-α) [5] to the EGFR leads to receptor dimerization and autophosphorylation [6]. The autophosphorylation of the EGFR at tyrosine residues activates downstream signaling, such as the Ras-Raf-MEK-p44/p42 mitogen-activated protein (MAP) kinase pathway or phosphotidylinositol-3 kinase (PI3K)-Akt pathway, thus resulting in the activation of cell proliferation [7]. The contribution of the EGFR pathway to oncogenesis has been well documented, and therapeutic exploitation of this axis has proven to be successful for several types of cancers, including colorectal and head and neck cancers [8,9]. The EGFR has been reported to be overexpressed in pancreatic cancer [10,11]. Therefore, EGFR activation appears to have a pivotal role in the growth and progression of pancreatic cancer, and EGFR-mediated pathways appear to be important potential targets for new therapies for this malignancy. The addition of EGFR-targeted therapy to gemcitabine in advanced pancreatic cancer has recently been demonstrated to provide a small, but statistically significant, survival benefit [12].

Rho GTPases are small proteins that act as molecular switches in a wide range of signaling pathways [13]. Three main classes of Rho GTPases, Rho, Rac and Cdc42, are known to regulate actin cytoskeletal dynamics [14]. Rho-associated coiled-coil containing protein kinase (Rho-kinase/ROCK) was initially characterized for its role in mediating the formation of RhoA-induced stress fibers and focal adhesion through its effects on the phosphorylation of the myosin light chain [15]. ROCK also phosphorylates LIM kinases 1 and 2 (LIMKs), which phosphorylate cofilin [14]. The phosphorylation of cofilin by LIMKs inactivates its actin-depolymerization activity [16]. Therefore, the phosphorylation of LIMKs by ROCK inhibits cofilin-mediated actin-filament disassembly and leads to an increase in the number of actin filaments [14]. It has been reported that the Rho-ROCK pathway plays an important role in various cellular functions such as vascular smooth muscle cell contraction, cell migration and cell proliferation [17].

Itoh *et al. *first reported that the expression of constitutively active ROCK promotes cell invasion, and that a ROCK inhibitor, Y27632 [18], reduces tumor cell dissemination *in vivo *[19]. An elevated expression of RhoA, as well as the Rho effector protein ROCK, are commonly observed in human cancers and often associated with more invasive and metastatic phenotypes [20]. In addition, the expression of ROCK1 is almost always found in pancreatic cancer tissues, but not in normal pancreatic tissues [21]. On the other hand, a recent report showed that Indole-3-carbinol (I3C), a phytochemical derived from cruciferous vegetables, decreased the metastatic spread of tumors in experimental animals in a ROCK-dependent manner [22]. In this report, I3C stimulated the phosphorylation of cofilin by activated ROCK, and inhibition of ROCK ablated the I3C-induced stress fiber formation and peripheral focal adhesion, which led to the inhibition of cell motility in human breast cancer cells [22].

We have recently reported that Rho-kinase/ROCK negatively regulates EGF-stimulated colon cancer cell proliferation [23]. Moreover, we have demonstrated that a Rho-kinase/ROCK inhibitor upregulates migration by altering focal adhesion formation via the Akt pathway in colon cancer cells [24], thereby indicating that ROCK might be considered as a new therapeutic target for colon cancer patients. However, the precise role of ROCK in cancer cells remains to be clarified. In the present study, we investigated the role of ROCK in the activation of the EGFR and subsequent cell proliferation pathway in pancreatic cancer cells.

## Materials and methods

### Materials

The ROCK inhibitor Y27632 was obtained from Calbiochem-Novabiochem Co. (La Jolla, CA). EGF and TGF-α were obtained from Sigma Chemical Co. (St. Louis, MO) and R&D Systems (Minneapolis, MN), respectively. Antibodies against total EGFR and glyceraldehyde-3-phosphate dehydrogenase (GAPDH) were obtained from Santa Cruz Biotechnology (Santa Cruz, CA). Antibodies against phospho-cofilin, cofilin, phospho-myosin light chain (MLC), phospho-EGFR (Tyr1045 and Tyr1068), phospho-MEK1/2, MEK1/2, phospho-p44/p42 MAP kinase, p44/p42 MAP kinase, phospho-Akt, Akt, phospho-glycogen synthase kinase (GSK)-3β, GSK-3β and ROCK1 were obtained from Cell Signaling, Inc. (Beverly, MA). Anti-EGFR-neutralizing antibodies were purchased from Millipore (Temecula, CA). The ECL Western blot detection system was purchased from GE Healthcare (Buckinghamshire, UK). Other materials and chemicals were obtained from commercial sources.

### Cell culture

Panc1, KP3 and AsPc1 pancreatic cancer cells were grown in Roswell Park Memorial Institute (RPMI) 1640 (Invitrogen, San Diego, CA) medium supplemented with 10% heat-inactivated fetal calf serum (FCS), penicillin (100 U/ml) and streptomycin (100 μg/ml) in a humidified 5% CO_2 _incubator at 37°C. Unless otherwise indicated, the cells were incubated in serum free medium for 24 h prior to each experiment.

### Cell proliferation assay

We used Cell Proliferation ELISA (BrdU) and a 3-(4,5-dimethylthiazol-2-yl)- 2,5-diphenyltetrazolium bromide (MTT) cell proliferation kit I (Roche Diagnostics Co., Indianapolis, IN) in accordance with instructions of the manufacturer. In the BrdU incorporation assay, Panc1, KP3 and AsPc1 cells were seeded onto 96-well plates (5 × 10^3 ^cells/well) in RPMI containing 10% FCS, and 48 h later, the cells were pretreated with 3 μM Y27632 or vehicle in RPMI containing 0.3% FCS for 1 h, and then stimulated with 30 ng/ml of EGF or vehicle for 24 h. In the MTT assay, Panc1 cells were seeded onto 96-well plates (3 × 10^3 ^cells/well), and 24 h later, the cells were treated with the indicated doses (0, 1 and 3 μM) of Y27632 in RPMI containing 3% FCS for 72 h. In the EGFR blockade experiments, Panc1 cells were seeded onto 96-well plates (1.5 × 10^3 ^cells/well) and 24 h later, the cells were treated with 0.5 μg/ml of anti-EGFR-neutralizing antibodies or normal mouse-IgG in RPMI containing 3% FCS for 0-4 days. The medium and agents were not changed during these periods. The remaining cells were counted by the MTT cell proliferation kit I. All assays were done in triplicate.

### The measurement of RhoA activity

RhoA activity was measured using G-LISA™ Small G-protein Activation Assays (Cytoskeleton, Denver, CO). In brief, the cells stimulated with EGF (30 ng/ml) for the indicated times were harvested using the lysis buffer contained in the kit. The cell lysates were then analyzed by FUJIFILM LAS-4000 multicolor (Tokyo, Japan).

### Western blot analysis

The cells were lysed in lysis buffer [20 mM Tris (pH 7.5), 150 mM NaCl, 1 mM EDTA, 1 mM EGTA, 1% TritonX-100, 2.5 mM sodium pyrophosphate, 50 mM NaF, 50 mM HEPES, 1 mM Na_3_VO_4 _and 2 mM phenylmethylsulfonyl fluoride (PMSF)] and scraped from the dishes. Protein extracts were examined by a Western blot analysis as previously described [25]. The proteins were fractionated and transferred onto an Immune-Blot PVDF Membrane (Bio-Rad, Hercules, CA). The membranes were blocked with 5% fat-free dry milk in phosphate-buffered saline (PBS) containing 0.1% Tween-20 (PBS-T) for 30 min before incubation with the indicated primary antibodies. Peroxidase-labeled antibodies raised in goats against rabbit IgG were used as secondary antibodies. The peroxidase activity on the membrane was visualized on X-ray film by means of the ECL Western blot detection system.

### siRNA protocol

We used two types of negative controls provided by Life Technologies (Japan; *Silencer*^® ^Negative Control #1 and #2 siRNA). Predesigned siRNAs targeting ROCK1 (ON-TARGET, J-003536-06, J-003536-07) were obtained from Thermo Scientific Inc. (Waltham, MA). The sequences were as follows; UAGCAAUCGUAGAUACUUA (J-003536-07, simplified as #1) and CUACAAGUGUUGCUAGUUU (J-003536-06, simplified as #2). Transfection was performed according to the manufacturer's protocol (Bio-Rad, Tokyo, Japan). In brief, 5 μl of siLentFect (Bio-Rad) and 10 nM of siRNA were diluted with FCS-free Opti-MEM, pre-incubated at room temperature for 20 min, and then added to Opti-MEM without FCS. The cells were incubated at 37°C for 24 h with siRNA-siLentFect complexes, and the medium was changed to fresh medium with 10% FCS, and cells were incubated for an additional 24 h. They were then treated as indicated, and subsequently harvested for the Western blot analysis.

### Immunofluorescence microscopy studies

Immunofluorescence microscopy studies were performed as described previously [26]. In the ROCK inhibition experiments using Y27632, the cells grown on coverslip-bottom dishes were treated with 3 μM Y27632 or vehicle for 1 h at 37°C, followed by exposure to 30 ng/ml of EGF for 10 min at 37°C. They were then fixed with 4% paraformaldehyde for 10 min on ice and then exposed to 0.1% Triton X-100 for 10 min to permeabilize the cell membrane. Next, they were exposed to anti-MLC antibodies, followed by exposure to Alexa Fluor 488^®^-conjugated goat anti-rabbit IgG antibodies and 4',6-diamidino-2-phenylindole (DAPI; Wako, Tokyo, Japan) for 1 h. In the EGFR-localization experiments, the cells were treated with 3 μM Y27632 or vehicle for 1 h at 37°C, and then labeled for 15 min at 37°C with anti-EGFR antibodies which recognize the extracellular domain of the EGFR. They were then exposed to 30 ng/ml of EGF for 10 min at 37°C. To observe only the cell surface EGFR that remained on the plasma membrane, these cells were not permeabilized. They were fixed and exposed to Alexa Fluor 488^®^-conjugated donkey anti-goat IgG antibodies and DAPI for 1 h, and then examined by fluorescence microscopy using a BIOREVO system (BZ-9000; Keyence, Tokyo, Japan) according to the manufacturer's protocol.

### Image analysis

The protein band intensities in the Western blot analysis were determined by integrating the optical density over the band area (band volume) using the NIH image software program. Based on the intensity of the control protein band on the X-ray film, the protein samples were quantitatively compared. The fluorescence intensity of the cell surface EGFR-labeled Alexa 488 was also measured and quantified using this software program.

## Results

### Effects of Y27632 on cell proliferation in Panc1, KP3 and AsPc1 pancreatic cancer cells

**Figure 1:**
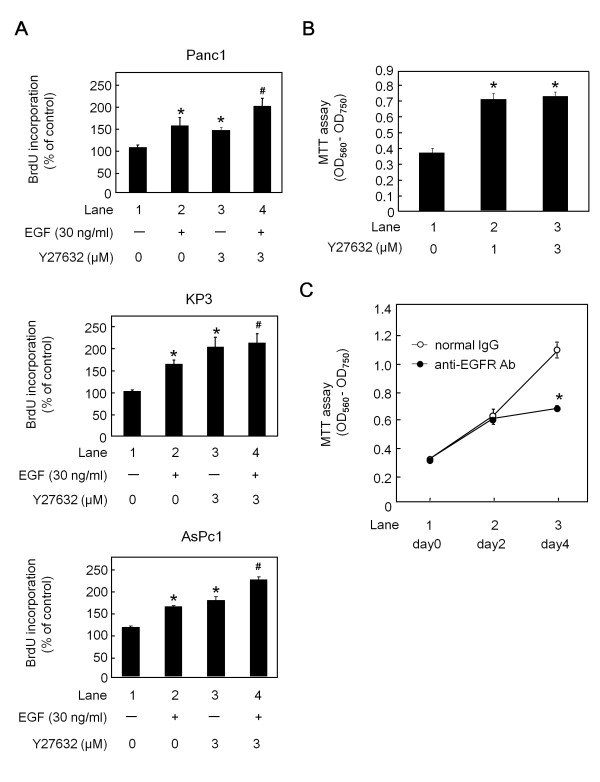
**The effects of Y27632 and EGF on pancreatic cancer cell proliferation**. (A) Panc1, KP3 and AsPc1 cells were pretreated with 3 μM Y27632 or vehicle in RPMI containing 0.3% FCS for 1 h, and then stimulated with 30 ng/ml of EGF or vehicle for 24 h. The BrdU incorporation as the % of the control (lane 1) is shown. (B) Panc1 cells were treated with the indicated doses of Y27632 or vehicle in RPMI containing 3% FCS for 48 h, and the cell viability assay was performed using the MTT cell proliferation kit I. (C) The attached cells were treated with 0.5 μg/ml of normal mouse-IgG (open circle) or anti-EGFR-neutralizing antibodies (closed circle) for the indicated periods in medium containing 3% FCS, and the surviving cells were counted using the MTT cell proliferation kit I. The results are expressed as the absorbance (OD 560 nm-OD 750 nm). All assays were done in triplicate. (*) indicates a significant difference (p < 0.05) compared with lane 1, (#) indicates a significant difference (p < 0.05), compared with lane 2.

In order to examine whether or not EGF and ROCK are involved in pancreatic cancer cell proliferation, we first evaluated BrdU incorporation in Panc1, KP3 and AsPc1 cells utilizing Y27632 as a specific ROCK inhibitor. When these cells were treated with EGF (Figure 1A, lane 2, respectively), the BrdU incorporation was increased. Interestingly, BrdU incorporation was also increased when these cells were treated with Y27632 alone (Figure 1A, lane 3 compared to lane 1, respectively). In addition, the BrdU incorporation induced by EGF was further enhanced when these cells were pretreated with Y27632 (Figure 1A, lane 4 compared to lane 2, respectively). To verify these results, we also performed another experiment using the MTT assay. The growth of Panc1 cells was significantly enhanced when the cells were pretreated with Y27632 at a dose over 1 μM (Figure 1B). Taken together, these results indicate that ROCK plays a suppressive role in pancreatic cancer cell proliferation.

### Effects of anti-EGFR-neutralizing antibodies on Panc1 pancreatic cancer cell proliferation

We next examined the effect of the blockade of EGF stimulation on the proliferation of Panc1 cells grown in medium containing 3% FCS. When the cells were treated with anti-EGFR-neutralizing antibodies for 4 days, the cell growth was significantly suppressed, compared to the cells treated with normal IgG (Figure 1C). Since medium containing 3% FCS is recognized to contain various types of growth factors, including EGF, it is likely that EGF stimulation plays an important role in Panc1 cell proliferation. These results led us to further investigate the role of ROCK in EGF-treated pancreatic cancer cells.

### Effects of EGF on RhoA activity and the phosphorylation of cofilin, MLC, and the EGFR at tyrosine residues in Panc1 cells

**Figure 2:**
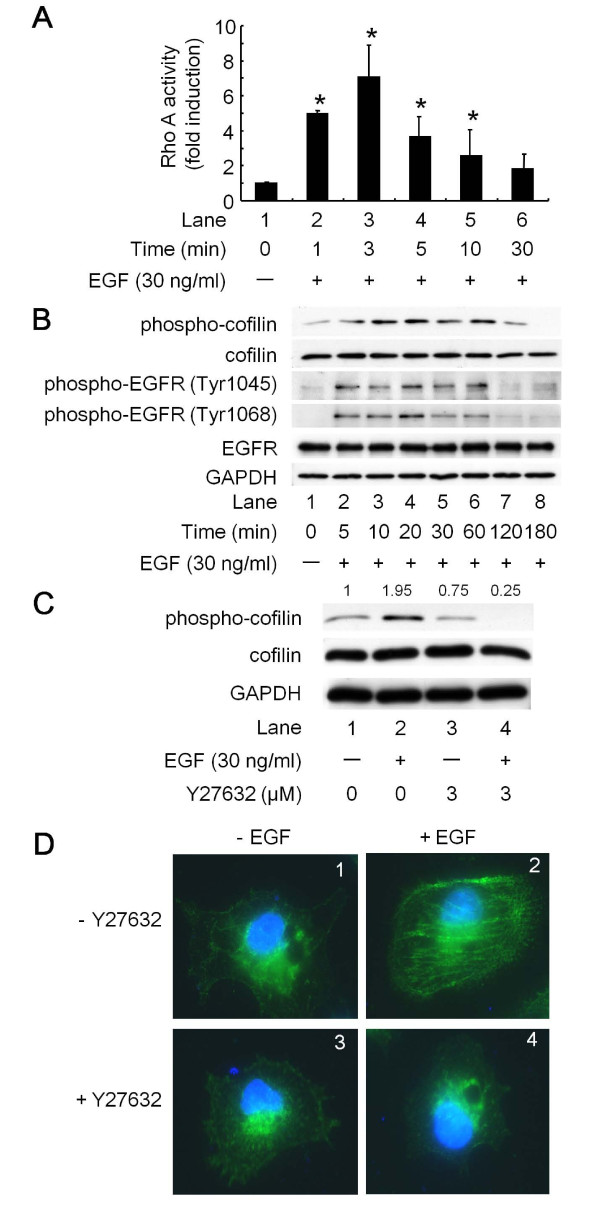
**The effects of EGF on RhoA activity and the phosphorylation of cofilin, MLC and EGFR at tyrosine residues in Panc1 cells**. (A) Panc1 cells were stimulated with 30 ng/ml of EGF for the indicated periods, and RhoA activity was measured by G-LISA™ Small G-protein Activation Assays. The results are expressed as the fold increase compared with untreated control cells. Bars designate SD of triplicate assays. (*) indicate significant increases (p < 0.05) compared to control cells. (B) Panc1 cells were stimulated with 30 ng/ml of EGF for the indicated periods. (C) Panc1 cells were pretreated with 3 μM Y27632 or vehicle for 1 h, and stimulated with 30 ng/ml of EGF or vehicle for 10 min. The cell lysates were then harvested and Western blotting were performed with antibodies against phospho-cofilin, cofilin, phospho-EGFR (Tyr1045 and Tyr1068), EGFR and GAPDH. The intensities of protein bands were determined by integrating the optical density over the band area using the NIH image software program. The intensity of each protein band was divided by the control (lane 1), and is shown above each panel. (D) Panc1 cells were treated with 3 μM Y27632 or vehicle for 1 h, followed by exposure to 30 ng/ml of EGF or vehicle for 10 min. After fixation, they were exposed to anti-phospho MLC antibodies, Alexa Fluor 488^®^-conjugated goat anti-rabbit IgG antibodies and DAPI. The cells were examined by fluorescence microscopy.

It is well known that EGF activates RhoA in many cell systems [27]. In order to elucidate the involvement of EGF in ROCK activation in Panc1 cells, we first examined the effect of EGF on RhoA activity in Panc1 cells. As shown in Figure 2A, 30 ng/ml of EGF significantly activated RhoA. The maximum effect was observed within 3 min and it continued for up to 10 min, and then decreased thereafter. These results suggest that EGF stimulation affects ROCK through RhoA.

It is generally recognized that cofilin is one of downstream substrates of ROCK, indicating that phosphorylation of cofilin reflects the activation of ROCK [14]. Moreover, EGF markedly induced the phosphorylation of cofilin in a time-dependent manner (Figure 2B). The effect of EGF on the phosphorylation of cofilin appeared at 5 min, reached a maximum at 10-20 min, and decreased at 180 min after EGF-treatment (Figure 2B, first panel). EGF also markedly and immediately induced the phosphorylation of EGFR at Tyr1045 and Tyr1068 at 0.5 min, reached a maximum within 1 min (data not shown), continued for up to 60 min, and decreased at 120 min after EGF-treatment (Figure 2B, 3rd and 4th panels). These results indicate that the activation of EGFR induced by EGF preceded the phosphorylation of cofilin, which reflects the activation of ROCK in Panc1 cells.

We next examined whether Y27632 inhibits the EGF-induced phosphorylation of cofilin. We observed that EGF induced the phosphorylation of cofilin (Figure 2C; upper panel, lane 2 compared to lane 1), and 3 μM of Y27632 completely suppressed the EGF-induced phosphorylation of cofilin (Figure 2C; upper panel, lane 4 compared to lane 2). Interestingly, Y27632 alone did not suppress the phosphorylation of cofilin at the basal level (Figure 2C; upper panel, lane 3 compared to lane 1).

The phosphorylation of MLC plays a critical role in controlling actomyosin contractility in smooth muscle and non-muscle cells [28], and ROCK has been reported to directly phosphorylate MLC *in vitro *[29]. To confirm that EGF activates ROCK in Panc1 cells, we examined the effects of EGF on the phosphorylation of MLC in an immunofluorescence microscope study. When the cells were stimulated with 30 ng/ml of EGF for 10 min, phosphorylated MLC was clearly observed in the cells (Figure 2D, panel 2 compared to panel 1). Moreover, pretreatment with 3 μM Y27632 markedly reduced the EGF-induced MLC phosphorylation (Figure 2D, panel 4 compared to panel 2). Taken together, these data strongly suggest that EGF induces the activation of ROCK via RhoA, and that the phosphorylation of cofilin and MLC by EGF occurs through ROCK in Panc1 pancreatic cancer cells.

### Effects of Y27632 on the phosphorylation of EGFR at tyrosine residues in Panc1, KP3 and AsPc1 pancreatic cancer cells

**Figure 3:**
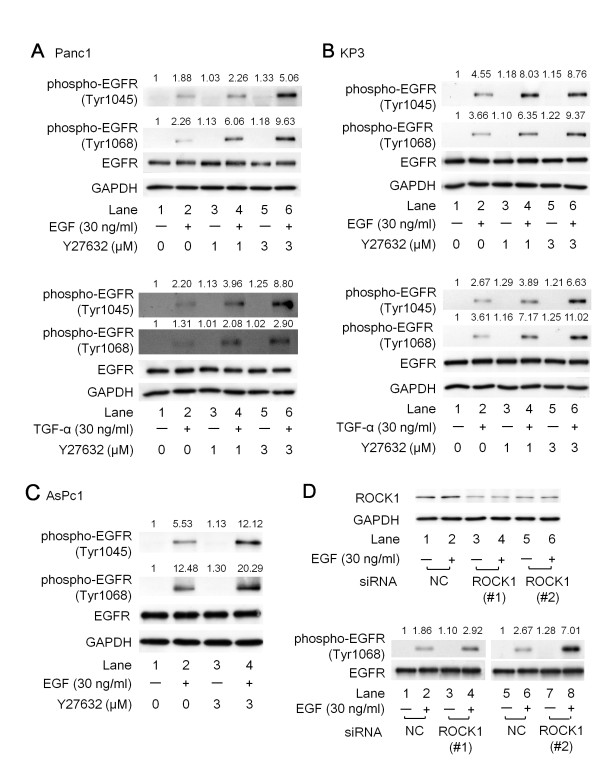
**The effects of Y27632 or ROCK1-siRNA on either EGF or TGF-α-induced phosphorylation of EGFR at tyrosine residues**. (A-C) Panc1 (A), KP3 (B) and AsPc1 (C) cells were pretreated with the indicated concentrations of Y27632 or vehicle for 1 h, and then stimulated with 30 ng/ml of EGF or 30 ng/ml of TGF-α or vehicle for 5 min. The cell lysates were then harvested and Western blotting were performed with antibodies against phospho-EGFR (Tyr1045 and Tyr1068), EGFR and GAPDH. (D) Upper panels; Panc1 cells were transfected with 10 nM of a negative control siRNA or siRNAs specifically targeting ROCK1 (#1 and #2) for 48 h, and then stimulated with 30 ng/ml of EGF or vehicle for 5 min. The cell lysates were then harvested and Western blotting was performed with antibodies against ROCK1, phospho- EGFR (Tyr1068), EGFR and GAPDH. The intensities of protein bands in the Western blot analysis were determined by integrating the optical density over the band area using the NIH image software program. The intensity of each protein band was divided by the control (lane 1), and is shown above each panel.

The EGFR is a transmembrane glycoprotein with an extracellular ligand-binding domain [30]. Binding of specific ligands such as EGF and TGF-α to the extracellular domain results in EGFR dimerization and autophosphorylation of the tyrosine kinase domain, leading to the activation of downstream signaling pathways that are involved in cell proliferation and survival [31]. We next examined the effects of Y27632 on the EGF-induced phosphorylation of EGFR at Tyr1045 and Tyr1068 in Panc1, KP3 and AsPc1 cells. Pretreatment of the cells with Y27632 for 1 h caused a significant increase in the phosphorylation levels of EGFR at Tyr1045 and Tyr1068 (Figure 3A, B and 3C). We also examined the effects of Y27632 on the TGF-α-induced phosphorylation of EGFR at Tyr1045 and Tyr1068 in Panc1 and KP3 cells, and obtained similar results to those obtained using EGF (Figures 3A and 3B; lower panels, respectively).

There are two isoforms of ROCK, known as ROCK1 and ROCK2, that share 65% overall homology at the amino acid level [32]. The tissue distribution of ROCK1 and ROCK2 is similar, and relatively few studies have delineated the specific roles of each ROCK [33]. To further verify that the inhibition of ROCK enhances EGF-induced phosphorylation of EGFR at tyrosine residues, we performed siRNA experiments, in which we transfected into Panc1 cells with negative control (NC)-siRNA or siRNA specifically targeting ROCK1. We verified that 10 nM of the siRNAs specifically targeting ROCK1 (both #1 and #2) caused 70% protein knockdown whereas 10 nM of the NC-siRNA did not affect the protein level of ROCK1 (Figure 3D; upper panels). We also confirmed this using another NC-siRNA (see Materials and Methods; data not shown). As expected, knockdown of ROCK1 caused significant enhancement of the phosphorylation levels of the EGFR at tyrosine residues (Figure 3D; lower panels), which is consistent with our results shown in Figures 3A-C.

### Effects of Y27632 on the EGF-induced phosphorylation of MEK1/2, p44/p42 MAP kinase, Akt and GSK-3β in Panc1 pancreatic cancer cells

**Figure 4:**
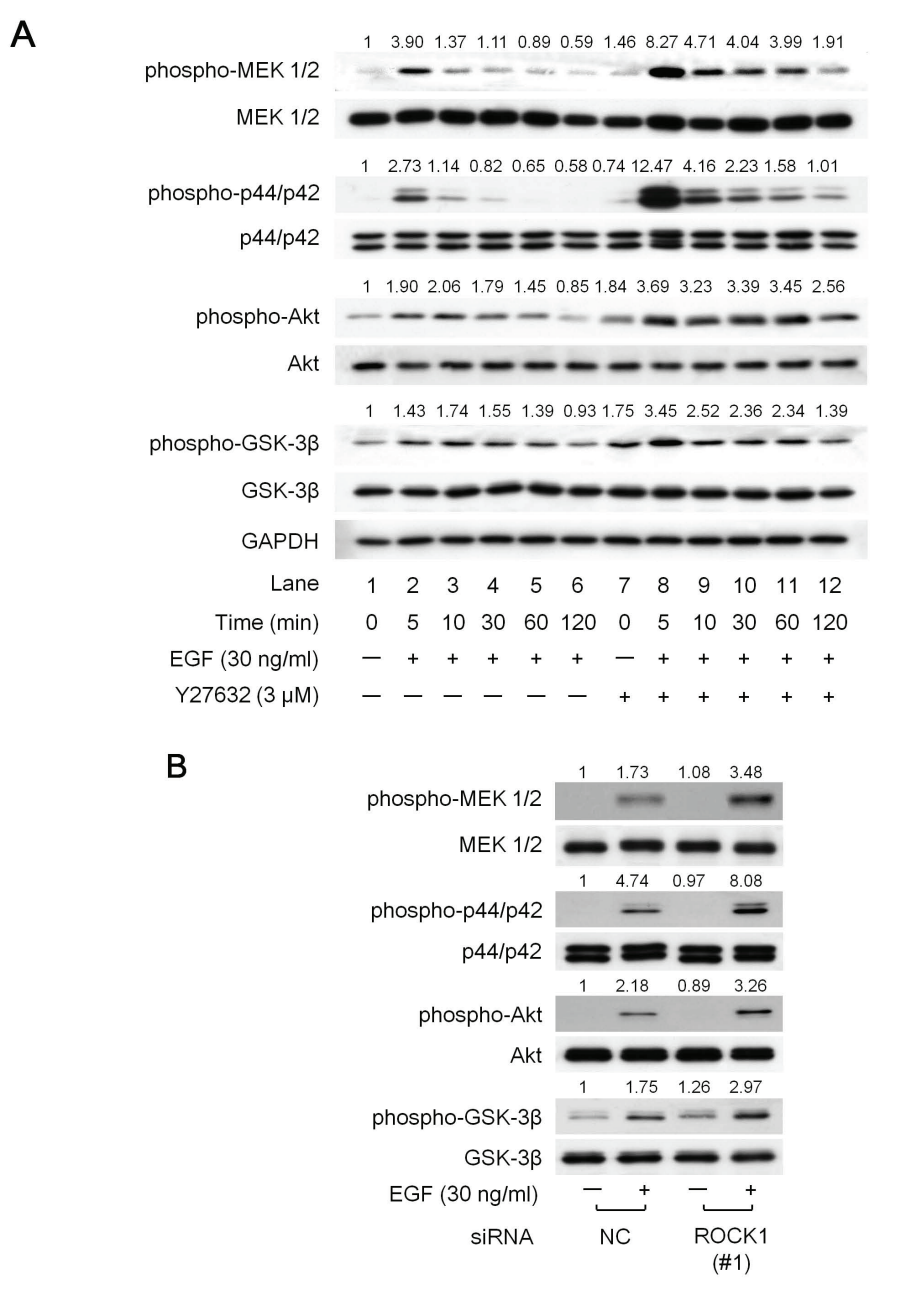
**The effects of Y27632 or ROCK1-siRNA on EGF-induced phosphorylation of MEK1/2, p44/p42 MAP kinase, Akt and GSK-3β in Panc1 cells**. (A) The cells were pretreated with 3 μM Y27632 or vehicle for 1 h, and then stimulated with 30 ng/ml of EGF or vehicle for the indicated periods. (B) The cells were transfected with 10 nM of a negative control siRNA or siRNAs specifically targeting ROCK1 (#1) for 48 h, and then stimulated with 30 ng/ml of EGF or vehicle for 5 min. The cell lysates were then harvested and Western blotting were performed with antibodies against phospho-MEK1/2, MEK1/2, phospho-p44/p42 MAP kinase, p44/p42 MAP kinase, phospho-Akt, Akt, phospho-GSK-3β, GSK-3β and GAPDH. The intensities of protein bands in the Western blot analysis were determined by integrating the optical density over the band area using the NIH image software program. The intensity of each protein band was divided by the control (lane 1), and is shown above each panel.

Two of the better-understood EGFR signal transduction pathways involved in cell proliferation are the Ras-Raf-MEK-p44/p42 MAP kinase pathway and the PI3K/Akt pathway [7]. Since pretreatment with Y27632 enhanced the phosphorylation of the EGFR at tyrosine residues (Figures 3A, B and 3C), we next examined the effects of Y27632 on the EGF-induced phosphorylation of MEK1/2 and p44/p42 MAP kinase in Panc1 cells. The effects of EGF on the phosphorylation of MEK1/2 and p44/p42 MAP kinase started at 1 min (data not shown), reached a maximum within 5 min, and decreased thereafter (Figure 4A, lanes 1-6). As expected, the EGF-induced MEK1/2 and p44/p42 MAP kinase phosphorylation were markedly increased and prolonged when the cells were pretreated with 3 μM of Y27632 for 1 h (Figure 4A, lanes 7-12 compared to lanes 1-6).

We next examined the effects of Y27632 on the EGF-induced phosphorylation of Akt and GSK-3β in Panc1 cells, since GSK-3β is a critical downstream element of the PI3K/Akt pathway in EGFR signaling [34]. The effects of EGF on the phosphorylation of Akt and GSK-3β also reached a maximum within 5 min and decreased thereafter (Figure 4A, lanes 1-6). The EGF-induced phosphorylation of Akt and GSK-3β were also significantly enhanced when the cells were pretreated with 3 μM Y27632 for 1 h, although the increases were less dramatic than those for either MEK1/2 or p44/p42 MAP kinase. These findings agree with our results showing that pretreatment with Y27632 enhanced the phosphorylation of the EGFR at tyrosine residues (shown in Figure 3). Furthermore, knockdown of ROCK1 caused a significant enhancement of the phosphorylation levels of MEK1/2, p44/p42, Akt and GSK-3β (Figure 4B), which was also consistent with our results shown in Figure 4A.

### Effects of Y27632 on the internalization of the EGFR in Panc1 pancreatic cancer cells

**Figure 5:**
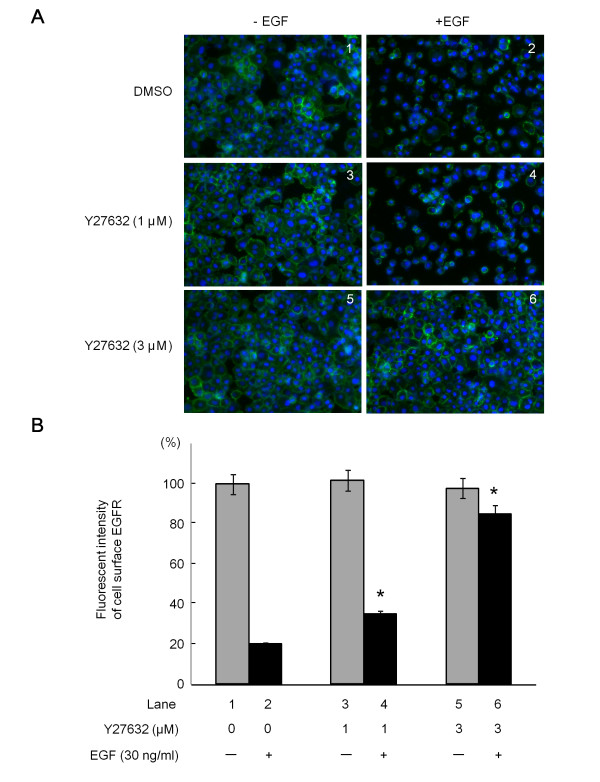
**The effects of Y27632 on the internalization of the EGFR in Panc1 pancreatic cancer cells**. (A) Panc1 cells were treated with the indicated concentrations of Y27632 or vehicle for 1 h at 37°C, and then labeled for 15 min at 37°C with anti-EGFR antibodies which recognize the extracellular domain of the EGFR. The cells were then exposed to 30 ng/ml of EGF for 10 min at 37°C. To observe only the cell surface EGFR that remained on the plasma membrane, these cells were not permeabilized with Triton X-100. They were then exposed to Alexa Fluor 488^®^-conjugated donkey anti-goat IgG antibodies and DAPI for 1 h. Representative cells examined by fluorescence microscopy are shown. (B) Green fluorescence signals from the remaining cell surface EGFR were measured using the NIH image software program, and expressed as the % of the control (compared with untreated control cells).

**Figure 6:**
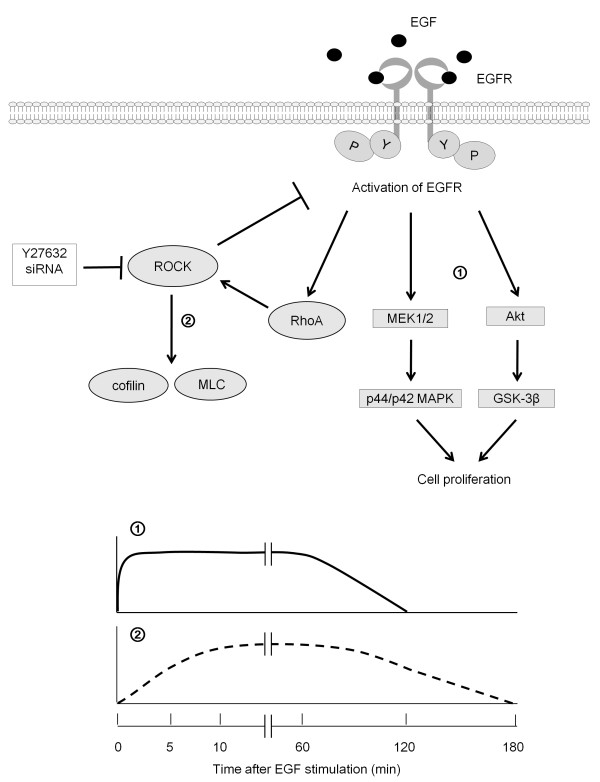
**A schematic representation of the involvement of ROCK in the EGFR signaling in pancreatic cancer cells**. First, EGF binds to the EGFR on the cell surface, and the EGFR undergoes dimerization and autophosphorylation at tyrosine residues. This triggers EGFR-related downstream signaling, such as activation of the MEK1/2-p44/p42 MAPK or Akt pathways, within 5 min of EGF stimulation (①). Around 5 min after the start of stimulation, EGF starts to induce the activation of RhoA and subsequently ROCK, as demonstrated by upregulation of the phosphorylation of cofilin and MLC (②). Inhibition experiments using Y27632 or siRNA indicated that activated ROCK cancels the persistent activation of the EGFR and the downstream pathways of MEK1/2 and Akt beginning at 5 min after the initiation of stimulation. Thus, ROCK functions to switch off the EGFR signaling that induces pancreatic cancer cell proliferation. EGF: epidermal growth factor, EGFR: EGF receptor, ROCK: Rho-associated coiled-coil containing protein kinase, MAPK: mitogen-activated protein kinase, GSK-3β: glycogen synthase kinase-3β, MLC, myosin light chain, P: phosphorylation, Y: tyrosine.

It is well known that EGF induces the internalization of the EGFR, and this is associated with subsequent ubiquitin-mediated degradation of the EGFR [35]. We showed in Figures 3 and 4 that Y27632 remarkably prolonged the EGF-induced activation of EGFR and subsequent signaling via MEK1/2 and Akt. Therefore, we next examined whether Y27632 affects the EGFR internalization by performing an immunofluorescence microscope study. In this assay, the cells were not permeabilized using Triton X-100 in order to observe the remaining EGFR on the cell surface. In unstimulated Panc1 cells, antibody-tagged EGFR was observed on the cell membrane (Figure 5A, panel 1), and the cell surface EGFR was significantly decreased when the cells were treated with EGF (Figure 5A, panel 2), which is consistent with our previous study [36]. Interestingly, when the cells were pretreated with increasing doses of Y27632, antibody-tagged EGFR still remained on the cells surface even after EGF stimulation for 10 min (Figure 5A, panels 4 and 6), while Y27632 alone had no effect on the localization of the EGFR (Figure 6A, panels 3 and 5). Quantification of the green fluorescence intensities of cell surface EGFR labeled with Alexa 488 revealed that the EGF-induced decrease in cell surface EGFR was restored by pretreatment with Y27632 in a dose-dependent manner (Figure 5B). These results strongly suggest that the inhibition of ROCK delayed the internalization of the EGFR induced by EGF in Panc1 cells.

## Discussion

In the present study, we investigated the role of ROCK in the proliferation of pancreatic cancer cells. We showed that Y27632, a specific ROCK inhibitor, enhanced cell proliferation (Figure 1A and 1B), thus suggesting a suppressive role of ROCK in pancreatic cancer cell proliferation. In addition, we found that EGF stimulation was necessary for cell growth in an experiment utilizing anti-EGFR-neutralizing antibodies to block EGFR signaling (Figure 1C). Therefore, we performed subsequent experiments focusing on the relationship between ROCK and EGF signaling. We demonstrated that EGF induced RhoA activity (Figure 2A), as well as the phosphorylation of both cofilin and MLC (Figure 2B), known downstream targets of ROCK [14]. We also demonstrated that Y27632 suppressed the phosphorylation of both molecules (Figure 2C and 2D), thus suggesting that the phosphorylation of cofilin and MLC by EGF occurs through ROCK in pancreatic cancer cells. We also found that the phosphorylation of the EGFR induced by EGF preceded the activation of ROCK (Figure 2B). Based on our findings, it is possible that the ROCK activation induced by EGF plays an inhibitory role in cell proliferation.

We next investigated the correlation between ROCK and EGF signaling, and found that exposure to Y27632 or knockdown of ROCK using siRNA strengthened the EGF-induced phosphorylation of EGFR at Tyr1045 and Tyr1068 (Figure 3). Moreover, the inhibition of ROCK activation using Y27632 or siRNA significantly augmented and prolonged the EGF-induced activation of MEK1/2 and p44/p42 MAP kinase, as well as Akt and GSK-3β (Figure 4). Taken together, our findings suggest that ROCK negatively regulates the EGFR pathway in pancreatic cancer cells. Moreover, Y27632 retarded the internalization of the EGFR induced by EGF (Figure 6), suggesting that ROCK is involved in the trafficking of the EGFR. While it has previously been reported that cell surface EGFR promotes cell growth, and that internalization of the EGFR induces cell death in breast cancer cells [37], our present findings suggest that cell surface EGFR retained after Y27632 treatment exerts pro-proliferative signals.

Our hypothetical pathway underlying ROCK-mediated signaling is summarized in Figure 6. After EGF binds to EGFR molecules on the cell surface, the receptor undergoes dimerization and autophosphorylation at tyrosine residues. This triggers EGFR-related downstream signaling, such as through the MEK1/2-p44/p42 MAP kinase or Akt pathways, within 5 min of stimulation (Figure 4). Around 5 min after stimulation is initiated, EGF starts to induce RhoA-mediated activation of ROCK, as demonstrated by up-regulation of cofilin and MLC phosphorylation (Figure 2). Inhibition experiments with Y27632 or siRNA indicated that ROCK suppresses the activation of the EGFR and the downstream pathways of MEK1/2 and Akt beginning at 5 min after the start of stimulation (Figures 3 and 4).

In addition to the elevated expression of RhoA, increased levels of ROCK have been reported to be observed in esophageal squamous cell carcinoma [38], bladder cancer [39] and pancreatic cancer [21]. In this study, the inhibition of ROCK prolonged EGFR signaling by preventing EGFR internalization, thus suggesting that ROCK functions to switch off the EGFR signaling by promoting EGFR desensitization. We speculate that the following occurs; 1) rapid growth of cancer cells results in overexpression of ROCK; 2) dysfunction of ROCK in cancer cells; 3) ROCK does not function in normal pancreatic cells, because they lack EGFR expression.

In addition, we transfected Panc1 cells with a ROCK1-encoding and examined the effect of EGF on EGFR phosphorylation in these cells. However, we did not observe any changes in the phosphorylation status of the EGFR induced by EGF between ROCK1-overexpressing cells and control cells (data not shown). This might be due to the fact that ROCK1 expression is already abundant in pancreatic cancer cells (Figure 3D). Taking all of this information into account, we speculate that ROCK1 is likely to be overexpressed in tumor tissues, and that it controls tumor invasion by a negative feedback system, because of the presence of excessive EGFR stimulation and the subsequent ROCK response in cancer cells. Moreover, we recently showed that ROCK suppressed Akt-dependent cell migration in colon cancer cells, thus suggesting that ROCK is also involved in metastatic events [24] in addition to cell proliferation.

With regard to the translation of this information to the clinic, appropriate regulation of ROCK might have the potential to be used as a new therapeutic target for human cancer, including pancreatic cancer, although further investigations are required to elucidate the exact mechanism(s) underlying how ROCK negatively regulates the activation of the EGFR.

## Conclusions

While EGF first stimulates the activation of EGFR, and subsequently induces pancreatic cancer cell proliferation, concurrent EGF-induced the activation of ROCK then turns off the activated EGFR pathway.

## Abbreviations

EGF: epidermal growth factor; EGFR: EGF receptor; TGF-α: transforming growth factor-α; ROCK: Rho-associated coiled-coil containing protein kinase; MAPK: mitogen-activated protein kinase; LIMKs: LIM kinases 1 and 2; GSK-3β: glycogen synthase kinase-3β; MLC: myosin light chain; MTT: 3-(4,5-dimethylthiazol-2-yl)- 2,5-diphenyltetrazolium bromide; P: phosphorylation; Y: tyrosine.

## Competing interests

The authors declare that they have no competing interests.

## Authors' contributions

SA designed the research studies; SA, MN, TY, TH and TY carried out the molecular biological studies; SA, IY, YO, OK and HM analyzed and interpreted the data; MN wrote the draft of the manuscript. All authors read and approved the final manuscript.
